# *In Vitro* Leishmanicidal Activities of Sesquiterpene Lactones from *Tithonia diversifolia* against *Leishmania braziliensis* Promastigotes and Amastigotes

**DOI:** 10.3390/molecules19056070

**Published:** 2014-05-14

**Authors:** Juliano S. de Toledo, Sergio R. Ambrósio, Carly H. G. Borges, Viviane Manfrim, Daniel G. Cerri, Angela K. Cruz, Fernando B. Da Costa

**Affiliations:** 1 Department of Cell Biology, Molecular and Pathogenic Bioagents, Faculty of Medicine of Ribeirão Preto, University of São Paulo (USP), Av. Bandeirantes 3900, Monte Alegre, Ribeirão Preto, SP 14049-900, Brazil; E-Mails: jstoledo@gmail.com (J.S.T.); manfrim.viviane@gmail.com (V.M.); dagcerri@gmail.com (D.G.C.); akcruz@fmrp.usp.br (A.K.C.); 2 Center for Research in Exact and Technological Sciences, University of Franca—UNIFRAN, Franca, SP 14404-600, Brazil; E-Mails: sergioambrosio@unifran.br (S.R.A.); carlyborges@gmail.com (C.H.G.B.); 3 AsterBioChem Research Team, Laboratory of Pharmacognosy, Department of Pharmaceutical Sciences, School of Pharmaceutical Sciences of Ribeirão Preto, USP, Av. do Café s/n, Ribeirão Preto, SP 14040-903, Brazil

**Keywords:** *Tithonia diversifolia*, Asteraceae, sesquiterpene lactones, antileishmanial activity, *Leishmania braziliensis*

## Abstract

Natural compounds represent a rich and promising source of novel, biologically active chemical entities for treating leishmaniasis. Sesquiterpene lactones are a recognized class of terpenoids with a wide spectrum of biological activities, including activity against *Leishmania* spp. In this work, a sesquiterpene lactone-rich preparation—a leaf rinse extract (LRE) from *Tithonia diversifolia*—was tested against promastigote forms of *L. braziliensis*. The results revealed that the LRE is a rich source of potent leishmanicidal compounds, with an LD_50_ value 1.5 ± 0.50 µg·mL^−1^. Therefore, eight sesquiterpene lactones from the LRE were initially investigated against promastigote forms of *L.* *braziliensis*. One of them did not present any significant leishmanicidal effect (LD_50_ > 50 µg·mL^−1^). Another had a cytotoxic effect against macrophages (4.5 µg·mL^−1^). The five leishmanicidal compounds with the highest level of selectivity were further evaluated against intracellular parasites (amastigotes) using peritoneal macrophages. Tirotundin 3-*O*-methyl ether, tagitinin F, and a guaianolide reduced the internalization of parasites after 48 h, in comparison with the negative control. This is the first report on sesquiterpene lactones that have potent leishmanicidal effects on both developmental stages of *L. braziliensis*.

## 1. Introduction

Leishmaniasis is a zoonosis caused by an intracellular parasite belonging to the genus *Leishmania*. *Leishmania* parasites have a dimorphic life cycle altering between an extracellular promastigote and an intracellular amastigote form [1]. This disease is endemic in 98 countries and an estimated 350 million people live in endemic areas [1,2]. In the absence of effective vaccines, the only means of treating and controlling leishmaniasis is chemotherapy. Pentavalent antimonials are the first-line drugs, but their high toxicity and the emergence of clinical resistance are obstacles to successful treatment [3,4]. The second-line drugs, such as amphotericin B and miltefosine, present other limitations, such as toxicity and exorbitant costs [4], which preclude their implementation in the public health systems of endemic countries. Therefore, the scientific community has been called to discover novel antileishmanial compounds with higher activity and fewer side effects [2,5]. Among the possibilities, compounds of natural origin (especially from the plant kingdom) represent a rich and promising source of novel, biologically active chemical entities [6,7,8,9].

*Tithonia diversifolia* (Hemsl.) A. Gray (Asteraceae), popularly known as Mexican sunflower or “margaridão”, has been used in folk medicine as an anti-inflammatory, and for treating diabetes, microbiological infections, snakebites, and malaria, among other ailments [10,11,12]. The plant’s major constituents are sesquiterpene lactones (STL) [10,11,13], which are recognized as a class of natural compounds with a wide spectrum of biological activities [14,15,16], including significant activity against tropical protozoan parasites [2,8,17]. Different STL subtypes have been reported as antileishmanial compounds. However, these studies have focused on other *Leishmania* species (*L. donovani*, *L. mexicana*, *L. major*, *L. infantum* or *L. amazonensis*) [8,18], and not *L. braziliensis*.

Based on the aforementioned lack of research, and as part of our ongoing efforts to discover novel natural compounds with antiparasitic properties [8,9,19,20], which include *in silico* studies [21], we report herein the significant antileishmanial activity displayed by *T. diversifolia* dichloromethane leaf rinse extract and the fact that its STL act against both the promastigote and amastigote forms of *L. braziliensis*.

## 2. Results and Discussion

**Figure 1:**
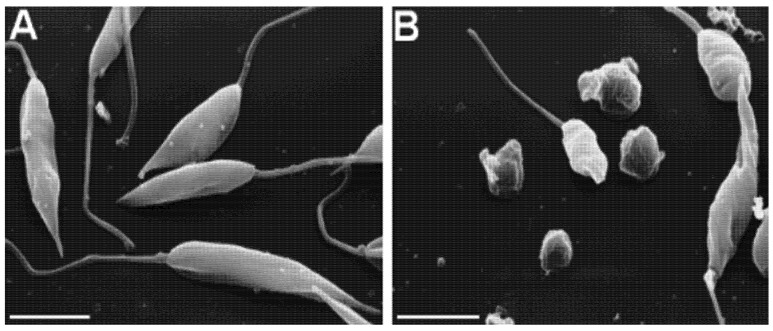
Scanning electron microscopy of untreated (**A**) and 10 µg/mL leaf rinse extract-treated (**B**) *L. braziliensis* promastigotes. Bar, 5 μm

We show, for the first time, that *T. diversifolia* dichloromethane leaf rinse extract (LRE) presents strong *in vitro* antileishmanial activity*.* Notably, LRE is a rich source of STL. During the rinse process (with organic solvents), these compounds are extracted from glandular trichomes located on the leaf surface; the trichomes are where STL are biosynthesized and stored [10,13]. The LRE showed a LD_50_ value of 1.5 ± 0.50 µg·mL^−1^. Through scanning electron microscopy (SEM), morphological analysis of promastigotes revealed noticeable differences between the treated parasites and the control group (Figure 1); treated parasites were rapidly exposed to LRE at 10 µg·mL^−1^ (6 h incubation). When parasites were incubated with LRE under these conditions, they lost two major promastigote characteristics: (i) their fusiform morphology changed to a rounded shape and; (ii) the flagellum in the majority of the cells was missing. All this information suggests that the LRE is a potential source of natural compounds with leishmanicidal activity. We accordingly decided to investigate the antileishmanial potential of the LRE’s main metabolites, *i.e.*, STL.

**Figure 2:**
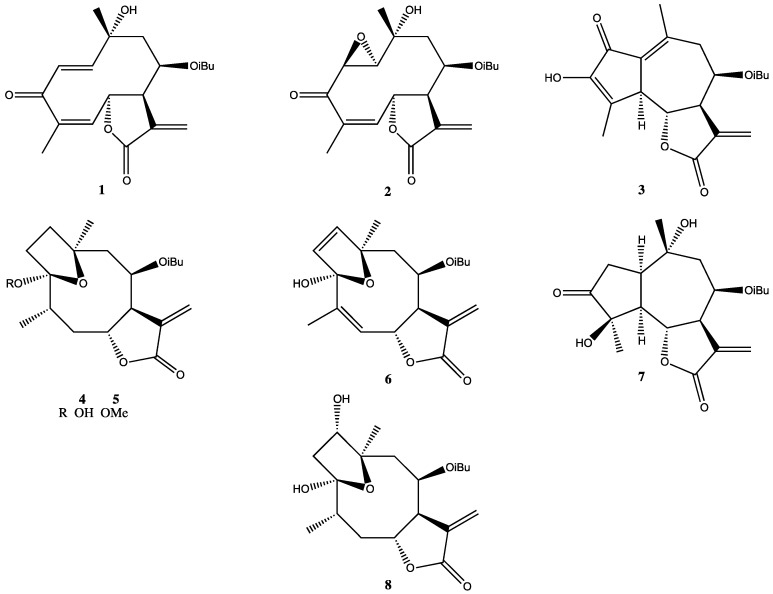
Chemical structures of sesquiterpene lactones isolated from the leaves of *T. diversifolia*.

Eight STL (Figure 2), which were previously isolated by our research group [13] from LREs, were investigated for activity against promastigote forms of *L.* *braziliensis* (Table). STL **3** was the only compound that did not show an *in vitro* leishmanicidal effect in the evaluated concentrations, and displayed an LD_50_ value that was higher than 50 µg·mL^−1^ (Table 1). Still, Compounds **1**, **2**, and **4**–**8** were very effective LD_50_ values ranged from 6.0 ± 2.5 to 37.4 ± 7.1 µM (see Table 1).

According to Schmidt *et al.* [18], the antiprotozoal activities displayed by STL correlate with their cytotoxicities, which are promoted by a Michael-type addition reaction of free thiol groups (usually from cysteine residues) from proteins with α,β-unsaturated carbonyls from the α-methylene-γ-lactone group. Cytotoxicity assays against macrophages for the effective compounds (Table 1) show that only Compound **1** (tagitinin C), the major STL present in LRE [13], causes significant cytotoxic effects, while displaying low selectivity (SI = 1.4). Interestingly, the chemical structure of **1** (Figure 2) has an α,β-unsaturated carbonyl group in the γ-lactone ring and a carbonyl group conjugated with two different double bonds in the germacrane ring; therefore, there are three reactive sites. On the other hand, the other STL, which have only one or two α,β-unsaturated carbonyl groups (compounds **4**–**8** and **2**, respectively), did not show significant toxicity in macrophages, and displayed a high level of selectivity (Table 1).

**Table 1:** *In vitro* antileishmanial activities of sesquiterpene lactones from *T. diversifolia* against *L. braziliensis* promastigotes and cytotoxic effects on peritoneal macrophages.

Compounds	LD_50_ for *Leishmania* µg·mL^−1^/µM	LD_50_ for macrophages µg·mL^−1^	Selectivity Index
**1**	3.2 ± 0.5/9.2 ± 1.4	4.5 ± 0.9	1.4
**2**	2.2 ± 0.9/6.0 ± 2.5	>50.0	>22.7
**3**	>50.0	>50.0	-
**4**	8.7 ± 1.9/24.7 ± 5.4	24.9 ± 1.1	2.9
**5**	13.7 ± 2.6/37.4 ± 7.1	>50.0	>3.6
**6**	7.4 ± 2.8/21.2 ± 8.0	>50.0	>6.7
**7**	9.0 ± 1.2/24.6 ± 3.3	>50.0	>5.5
**8**	7.5 ± 3.2/20.4 ± 8.7	>50.0	>6.6

During the blood meal intake, metacyclic promastigotes are regurgitated by the sandfly into the host’s skin. Metacyclic promastigotes bind to different receptors on phagocytic cells (neutrophils and macrophages) that are found in the sandfly’s bite site and are phagocytosed. Inside of parasitophorous vacuoles, metacyclic promastigotes transform into aflagellate amastigotes. The replication of these forms promotes the rupture of host cells, thus releasing infective amastigotes. This promotes the massive infection of macrophages, so as to continue the transmission cycle [1,22].

**Figure 3:**
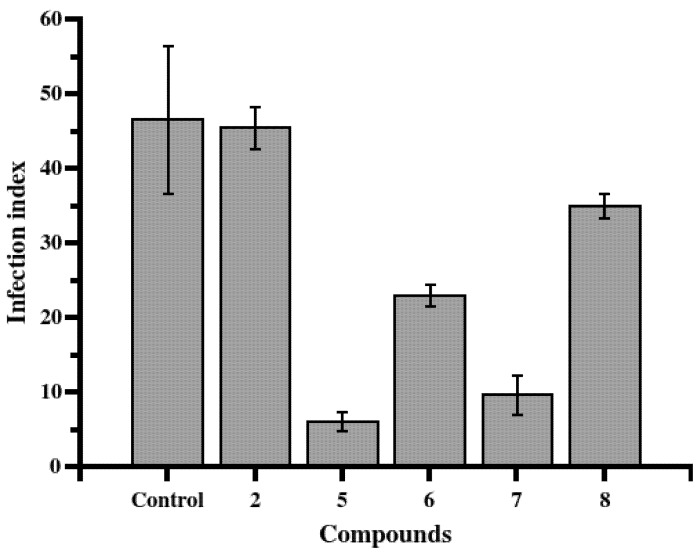
Leishmanicidal activity of STL **2** and **5**–**8** against intracellular parasites. Infected peritoneal macrophages were treated with STL at LD_50_ concentration, previously determined as for promastigotes, for 48 h at 34 °C and 5% of CO_2_. Compounds **5**, **6** and **7** showed statistically significant differences (*T*-test *p* values of 0.012, 0.035 and 0.018, respectively) when compared with control. The infection index was calculated by multiplying the percentage of infected macrophages by the average number of amastigotes per macrophage. Control corresponds to untreated infected peritoneal macrophages.

To better understand the antileishmanial potentials of the STL isolated from the LRE, we decided to assess these metabolites against intracellular parasites using peritoneal macrophages. For this purpose, the most effective compounds that showed the highest levels of selectivity (compounds **2** and **5**–**8**) were evaluated at their LD_50_ concentrations. The results showed that, when compared to negative control (untreated infected peritoneal macrophages), STL **5**, **6**, and **7** were able to reduce significantly (*p* < 0.05) the infection index after 48 h of treatment (Figure 3).

STL **5** and **6**, which belong to the heliangolide subtype, showed better effects than guaianolide **7** did. To the best of our knowledge, this is the first report of STL activity against both forms of *L. braziliensis* (*i.e.*, against both promastigotes and amastigotes).

## 3. Experimental

### 3.1. Plant Material, Extraction and Isolation of STL

*T. diversifolia* leaves were collected in May, 2002, by S.R.A. in Ribeirão Preto, SP, Brazil. Plant material was identified by Prof. J. N. Nakajima (Federal University of Uberlândia, MG, Brazil). A voucher specimen (FBC #126) was deposited at the SPFR herbarium (Department of Biology, FFCLRP, USP, Ribeirão Preto, SP, Brazil). Air-dried whole leaves (3.7 kg) were rapidly rinsed with CH_2_Cl_2_ to yield 82 g of light-yellow extract (LRE). Detailed information about the fractionation of the LRE via different chromatographic techniques and the purification of STL has been previously reported [13]. The LRE afforded tagitinin C (**1**, 88.0 mg), 1β,2α-epoxytagitinin C (**2**, 10.0 mg), 3-hydroxy-8β-isobutyryloxydehydroleucodin (**3**, 12.0 mg), tirotundin (**4**, 2.0 mg), tirotundin 3-*O*-methyl ether (**5**, 2.5 mg), tagitinin F (**6**, 4.0 mg), 4β,10α-dihydroxy-3-oxo-8β-isobutyryloxyguai-11(13)-en-6α,12-olide (**7**, 3.0 mg), and tagitinin A (**8**, 6.0 mg).

### 3.2. Parasites

Cultures *L. braziliensis* (H3227 MHOM/BR/94/H-3227) promastigotes were originally isolated from a human case of mucocutaneous leishmaniasis in the state of Ceará, Brazil. The cultures were maintained at 25 °C in M199 medium supplemented with 10% FBS (Fetal Bovine Serum), 2% human urine, 100 mM adenine, 10 mg·mL^−1^ hemin, 40 mM HEPES (pH 7.4), 50 units·mL^−1^ penicillin, and 50 mg·mL^−1^ streptomycin.

### 3.3. Macrophages

Thioglycolate-elicited peritoneal macrophages were obtained from BALB/c mice (weighing 20–25 g) by injection of 1 mL of 3% thioglycolate three days prior to peritoneal lavage with 10 mL of cold PBS (Phosphate-Buffered Saline; 137 mM NaCl, 8 mM Na_2_HPO_4_, 2.7 mM KCl, 1.5 mM KH_2_PO_4_, pH 7.0). The peritoneal exudate cells were centrifuged at 400 *×g* for 10 min and the pellet was incubated at 37 °C for 15 min in Ammonium-Chloride-Potassium (ACK) lysis solution (0.15 M NH_4_Cl, 10 mM KHCO_3_, 0.1 mM EDTA, pH 7.4) for removal of the remaining red blood cells. Macrophages were pelleted at 400 *×g* for 10 min, washed with PBS, and re-suspended in RPMI 1640 medium (Gibco BRL, Grand Island, NY, USA) supplemented with 10% heat-inactivated FBS and 1% streptomycin/penicillin at 1 × 10^6^ cells·mL^−1^.

### 3.4. Antileishmanial Assay

The antileishmanial assay was carried out according to the procedures outlined by Dutta *et al.* [23]. The LRE, as well as STL, were tested at the range of 50.0 to 0.12 µg·mL^−1^ in 96-well microplates in Schneider’s medium, which was supplemented with 10% FBS and 2% human urine. 4 × 10^4^ promastigote forms were seeded in each well (*i.e.*, 2 × 10^5^ parasites·mL^−1^) and the plate was incubated at 26 °C for 72 h. After that, 100 μg of 3-(4,5-dimethylthiazol-2-yl)-2,5-diphenyltetrazolium bromide (MTT), dissolved in 10 μL of sterile PBS, were added per well and the plates were incubated at 37 °C for 4 h. The plates were centrifuged at 4,000× *g* for 5 min and the supernatant was removed. The precipitated formazan was dissolved with 100 µL of dimethyl sulfoxide (DMSO) and absorbance was measured at 492 nm. The assays were evaluated in triplicate and repeated at least twice.

### 3.5. Antiamastigote Activity

The antiamastigote assay was carried out as previously described [24,25]. Briefly, a suspension of 5 × 10^6^ macrophages and 5 × 10^7^ parasites (10:1 parasites per cell ratio) in 10 mL RPMI 1640 medium were incubated at 27 °C in a polypropylene tube for 3 h. Non-internalized parasites were removed by three centrifugation cycles at 50 *×g*. The infected macrophages were plated on glass coverslips (13 mm diameter) in 24-well plates and incubated for 1 h at 34 °C; non-adherent macrophages were removed by washing (three times) with PBS. Infected macrophages were treated with STL **2** and **5**–**8** diluted in RPMI medium in the concentration previously determined as the LD_50_ for promastigotes and then incubated for 48 h at 34 °C in 5% CO_2_. Untreated infected macrophages were used as control. The coverslips were washed with PBS, stained with Panoptic stain (Diff-Quick; Baxter Scientific, Miami, FL, USA), dried and mounted on glass slides with Tissue-Tek mounting medium (Sakura Finetek Europe B.V., Alphen aan den Rijn, Holland). Three hundred macrophage cells per experiment were inspected through bright-field microscopy. The data was expressed as infection index (percentage of infected macrophages by the average number of amastigotes per macrophage) [24,25]. The tests were performed in triplicate with two independent experiments.

### 3.6. Cytotoxicity Assay

Macrophages (2 × 10^5^ cells/well) were seeded in a 96-well microtiter plate and incubated at 37 °C in a 5% CO_2_ atmosphere for 18 h. After that, the media were removed and adhered cells were exposed to the compounds (exposure concentrations ranged from 0.12 to 50.0 µg·mL^−1^) for 48 h under the same incubation conditions. The control wells without STL (untreated cells) were used as controls and were considered to be 100% viable cells. Cellular viability was determined using the MTT assay (as described for the antileishmanial assay). Each assay was performed in triplicate with two independent experiments.

### 3.7. Statistical Analysis

Data represent the mean number (±SD) of triplicate samples from two independent assays. The 50% lethal dose (LD_50_) values were calculated using dose–response curves in OriginLab software (OriginLab Corporation, Northampton, MA, USA).

### 3.8. Scanning Electron Microscopy

Promastigote (1 × 10^8^) forms of *L. braziliensis* were grown in the presence or absence of LRE from *T. diversifolia* (10 μg·mL^−1^ for 6 h). Cells were attached on 13 mm round coverslips previously coated with Biobond (Electron Microscopy Sciences, Hatfield, PA, USA) following the manufacture’s instructions. Cells were fixed in 2.0% glutaraldehyde (Ladd Research Industries, Burlington, VT, USA) for 2 h at RT. Next, they were submitted to a post-fixation process with 1% OsO_4_ (Electron Microscopy Sciences) for 2 h, rinsed in milli-Q water, and incubated with saturated thiocarbohydrazide (Electron Microscopy Sciences) for 10 min, followed again by 1% OsO_4_. This step was repeated twice. The promastigotes were dehydrated with a graded series of ethanol and were critically point-dried with liquid CO_2_ in a BAL-TEC-CPD 030 Critical-Point Dryer (BAL-TEC AG, Liechtenstein). Subsequently, the coverslips were mounted on aluminum stubs with silver paint (Electron Microscopy Sciences) and coated with gold in a BAL-TEC SCD 050 Sputter Coater (BAL-TEC). Finally, promastigotes were examined in a JEOL JSM-5200 scanning electron microscope (Jeol Ltd., Tokyo, Japan).

## 4. Conclusions

In this work, among seven leishmanicidal STL **1**, **2** and **4**–**8** from the LRE of *T. diversifolia*, three of them, namely two heliangolides **5** and **6** and one guaianolide **7**, showed potent effects against both developmental stages of *L. braziliensis* (*i.e.*, against both promastigotes and amastigotes). These three STL did not show considerable cytotoxicity when tested in peritoneal macrophages. *In vivo* efficacy studies of these active compounds and a better understanding of their mechanisms of action in *Leishmania* should be pursued. The results described herein bring new perspectives in the study of these two STL subtypes and may provide promising leads for the discovery of new agents against leishmaniasis.
